# The mediating effect of blood pressure between healthy lifestyles and stroke: Results from the China Kadoorie Biobank study

**DOI:** 10.7555/JBR.39.20250177

**Published:** 2025-08-29

**Authors:** Zidong Wang, Jiaxi Zhou, Xikang Fan, Jian Su, Houyue Geng, Xun Wu, Yujie Hua, Hongfu Ren, Jun Lyu, Pei Pei, Canqing Yu, Dianjianyi Sun, Yan Lu, Jinyi Zhou, Ran Tao

**Affiliations:** 1 School of Public Health, Nanjing Medical University, Nanjing, Jiangsu 211166, China; 2 Department of Noncommunicable Chronic Disease Control and Prevention, Jiangsu Provincial Center for Disease Control and Prevention, Nanjing, Jiangsu 210009, China; 3 Department of Noncommunicable Chronic Disease Control and Prevention, Suzhou City Center for Disease Control and Prevention, Suzhou, Jiangsu 215004, China; 4 Department of Noncommunicable Chronic Disease Control and Prevention, Wuzhong District of Suzhou City Center for Disease Control and Prevention, Suzhou, Jiangsu 215100, China; 5 Department of Epidemiology and Biostatistics, School of Public Health, Peking University, Beijing 100191, China; 6 Public Health and Epidemic Preparedness and Response Center, Peking University, Beijing 100191, China; 7 Key Laboratory of Epidemiology of Major Diseases, Ministry of Education (Peking University), Beijing 100191, China

**Keywords:** stroke, blood pressure, lifestyle, mediation analysis

## Abstract

While a healthy lifestyle is known to reduce the risk of stroke, the extent to which blood pressure (BP) mediates this association remains unclear. The present study aimed to quantify the mediating role of BP in the association between combined lifestyle factors and stroke incidence. Using data from 51929 participants free of major cardiovascular diseases or cancer at baseline, we employed structural equation modeling to assess the mediating effects of systolic (SBP) and diastolic (DBP) blood pressure. During the follow-up, 2811 incident stroke cases were identified. A healthy lifestyle was significantly associated with a reduced risk of stroke, with SBP mediating 44.70% (*β* = −0.0014, 95% confidence interval [CI]: −0.0016 to −0.0012) and DBP mediating 37.81% (*β* = −0.0012, 95% CI: −0.0015 to −0.0009) of this association. The mediating effects were attenuated but remained significant for ischemic stroke (SBP: 33.21%; DBP: 27.24%). In conclusion, approximately two-fifths of the protective association between a healthy lifestyle and stroke may be mediated by BP. These findings suggest that BP control may serve as an important early indicator for evaluating the effectiveness of lifestyle interventions in reducing stroke risk.

## Introduction

Stroke is a major cause of death and disability-adjusted life-years worldwide and imposes a particularly heavy burden in China^[1]^. It is well established that adherence to a healthy lifestyle is the primary strategy for stroke prevention^[2]^. Moreover, growing evidence supports the use of lifestyle interventions for preventing hypertension^[3–4]^, which is a key indicator of stroke^[5]^. However, whether and to what extent blood pressure (BP) mediates the association between lifestyle and stroke remains unclear.

Lowering BP could be a crucial step in the pathway between lifestyle interventions and stroke prevention^[5–6]^. Findings from the UK Biobank showed that, compared with a combination of healthy lifestyle factors, an unhealthy lifestyle increased the hazard ratio (HR) for incident stroke to 1.66 (95% confidence interval [CI]: 1.45 to 1.89)^[7]^. The Chinese Guidelines for the Prevention and Treatment of Hypertension emphasize that a healthy lifestyle, including a balanced diet, weight control, non-smoking, moderate alcohol consumption, and regular physical activity, is key to reducing hypertension risk^[4]^. Additionally, a meta-analysis of 54 randomized controlled trials (RCTs) found that a 10 mmHg decrease in systolic blood pressure (SBP) was associated with a 27% reduction in stroke incidence (relative risk [RR] = 0.73, 95% CI: 0.68 to 0.77)^[8]^. Moreover, a recent study developed models incorporating SBP and six other factors to predict the 10-year risk of stroke subtypes, demonstrating good discrimination^[9]^. In summary, BP may partially mediate the relationship between lifestyle interventions and stroke prevention.

In the present study, we used subcohort data from the China Kadoorie Biobank (CKB) study and employed a mediation analysis approach to quantify the extent to which BP mediated the association between combined lifestyle factors and stroke.

## Materials and methods

### Study population

**Figure 1:**
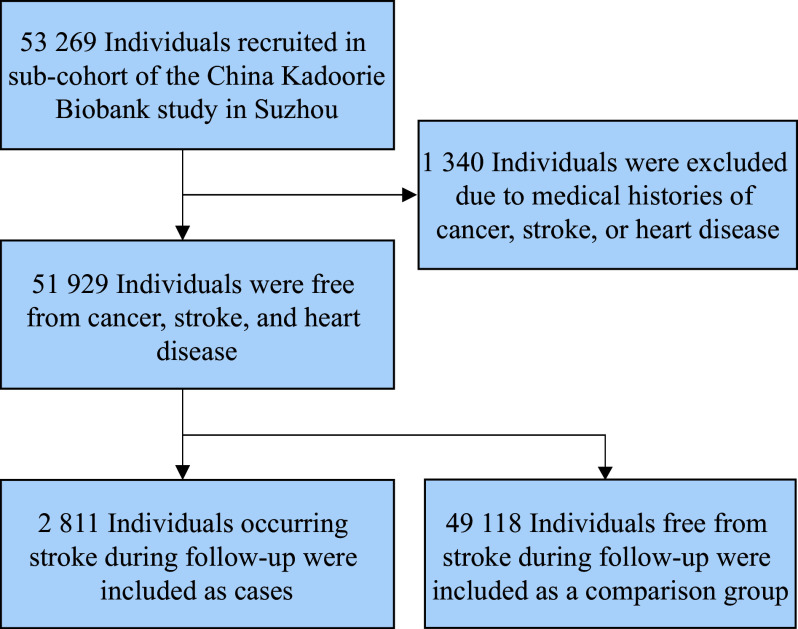
Flow chart of the study sample.

The CKB is a population-based prospective study. The data used in the present study originated from the Wuzhong District of Suzhou, one of the ten regions participating in the CKB. Overall, 53269 participants aged between 30 and 79 years were recruited between August 2004 and January 2008. Previous publications have described the CKB cohort in detail^[10]^. All participants completed questionnaires, underwent physical measurements, and provided informed consent. The CKB study was approved by the Ethical Review Committee of the Chinese Center for Disease Control and Prevention (Beijing, China) and the Oxford Tropical Research Ethics Committee (University of Oxford, UK). Written informed consent was obtained from all participants. All methods were carried out in accordance with the relevant guidelines and regulations. After excluding 1340 individuals with medical histories of stroke, heart disease, and cancer at baseline, 51929 individuals were included in the final analyses. A detailed description of the selection process is shown in ***Fig. 1***.

### Assessment of lifestyle factors

A range of lifestyle factors was obtained at baseline. Information on tobacco smoking status, type, frequency, the number of cigarettes smoked daily for current smokers, and the reason for quitting for former smokers. Alcohol consumption included typical drinking frequency and the volume of alcohol consumed on a typical drinking day over the past year. Physical activity information included the usual types of activity (occupational, commuting, domestic, and leisure-time) and the duration of activities in the past year. The daily level of physical activity was calculated based on the metabolic equivalent tasks (METs) for each activity by multiplying the MET value for each activity by the hours spent on it and summing the MET hours for all activities^[2,11]^. Habitual dietary intake during the past year was assessed using a qualitative food frequency questionnaire that included 12 common food groups in China. The questionnaire's relative validity and reproducibility were validated in previous studies^[12]^. Trained investigators measured weight using a body composition analyzer (TBF-300GS; Tanita Corp., Tokyo, Japan), and measured height and waist circumference (WC) using standard calibrated instruments. Body mass index (BMI) was calculated as weight (kg)/[height (m)]^2^.

### Definition of combined healthy lifestyle factors

Combined healthy lifestyle factors were defined by smoking, alcohol consumption, physical activity, habitual diet, and body shape^[2]^. For smoking, the healthy group included individuals who had never smoked or those who had quit without being affected by a disease^[2,13]^, because including those who quit due to illness may introduce bias. For alcohol consumption, the healthy group was defined as individuals who had never consumed alcohol, consumed alcohol weekly, or consumed a moderate amount of alcohol daily (*i.e.*, less than 25 g of pure alcohol per day for men and less than 15 g for women)^[13]^. The healthy physical activity group comprised individuals whose physical activity levels exceeded the median level of their respective age- (< 50 years, 50–59 years, and ≥ 60 years) and sex-specific groups. Habitual diet consisted of six food items based on the Chinese Dietary Guidelines and a previous study, including vegetables, fruits, eggs, red meat, grains, and fish^[14–16]^. A diet score was created using the following criteria: consuming vegetables and fruits daily, consuming eggs ≥ 4 days/week, consuming red meat 1–6 days/week, and consuming grains and fish every week. For each dietary item, participants who met the criterion received a score of 1; otherwise, they received a score of 0. Thus, the dietary score ranged from 0 to 6. Those with scores ≥ 4 were classified as the healthy group. In terms of body shape, both BMI and WC were considered. Participants with a moderate BMI (18.5 ≤ BMI < 24.0 kg/m^2^) and WC (WC < 85 cm for women and < 90 cm for men) were included in the healthy group^[2]^. The number of healthy lifestyle factors ranged from 0 (unhealthiest) to 5 (healthiest).

### Mediators

SBP and diastolic blood pressure (DBP) were treated as mediators in the analysis. BP was assessed by trained staff using a UA-779 digital monitor (A&D Company, Ltd., Tokyo, Japan), with at least two readings taken per participant. The mean of these readings was used as the BP value for each individual. To facilitate a direct comparison of effect sizes and enhance coefficient interpretability, these mean BP values were then standardized before being entered into the mediation models.

### Outcome

Incident stroke data were obtained from the local disease and death registration system, the medical insurance system, and active follow-up^[10]^. Stroke cases were coded by trained staff in accordance with the International Classification of Diseases 10^th^ Revision (ICD-10). Participants not covered by the medical insurance system were followed up annually to ascertain their hospital admissions, death, or migration out of the study area. All stroke events were reviewed and integrated by neurologists from China and the UK. Stroke was coded as I60–I61 or I63–I64; ischemic stroke (IS) was coded as I63.

### Covariates

Potential factors known to be associated with lifestyle, BP, and stroke were included as confounders in the analyses. These variables included self-reported age (entered as a linear term), sex (women and men), highest education level (no formal school, primary or middle school, and high school and above), household income (< 20000, 20000–34999, and ≥ 35000 RMB per year), family history of stroke or heart attack (yes, no, and unknown), duration of sedentary behavior (≤ 21 h/week, > 21 h/week), usage of antihypertensive drugs (yes or no), a history of diabetes (yes or no), respiratory disease (yes or no), kidney disease (yes or no), and digestive disease (yes or no). A family history of stroke or heart attack was defined as having at least one first-degree relative with these diseases. The duration of sedentary behavior was calculated by summing the hours spent watching TV or reading weekly. Diabetes included self-reported diabetes and screen-detected diabetes^[17]^. Screen-detected diabetes was defined as having a random blood glucose level ≥ 11.1 mmol/L or a fasting blood glucose level ≥ 7.0 mmol/L, measured using the SureStep Plus system (LifeScan, Inc., Milpitas, CA, USA)^[18]^.

### Statistical analysis

The characteristics of participants with combined lifestyle factors were presented as means (standard deviations [SDs]) for continuous variables or frequencies (percentages) for categorical variables. Pearson or Spearman correlation analyses were used to examine the association among lifestyle factors, BP, and all covariates. Given that most variables in the present study were self-reported by participants, Harman's single-factor test was performed to examine common method bias and to mitigate its potential impact. If the explanation rate of the largest common factor was less than 40%, it was considered that there was no serious common method bias in the study data^[19]^.

**Figure 2:**
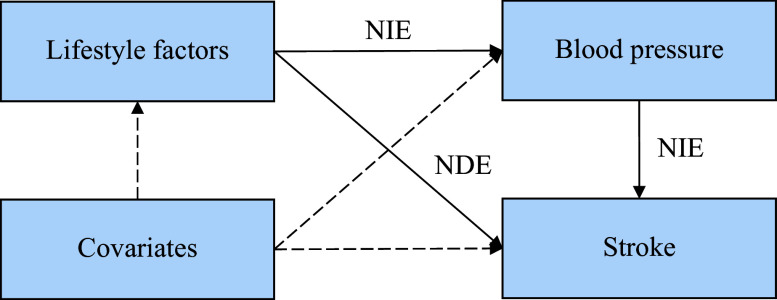
Mediating pathway of the association of lifestyle with stroke. Structural equation model of mediation of the association between lifestyle and stroke by blood pressure. Abbreviations: NDE, natural direct effect; NIE, natural indirect effect.

To elucidate the relationship between lifestyle and stroke, we conducted structural equation modeling (SEM) to quantify the natural indirect effects (NIEs) mediated by BP and the natural direct effects (NDEs) not mediated by BP (***Fig. 2***). We chose SEM for our mediation analysis over alternatives such as marginal structural models (MSMs), primarily because of our study design. MSMs are specifically designed to address the complex issue of time-varying confounding^[20]^, while our exposure (healthy lifestyle) and mediator (BP) were measured at a single baseline time point. In this context, SEM is a more appropriate and parsimonious choice, offering the key advantages of simultaneous estimation of all pathways and the assessment of overall model fit^[21]^, making it ideal for testing our hypothesized mediation model.

The SEM process encompassed two steps. First, a multivariate linear regression model was constructed to estimate the effect of lifestyle on BP. Second, a multivariate logistic regression model was constructed to model stroke as a function of lifestyle and BP. The NIEs indicated the effect of lifestyle on stroke explained by BP, while the NDEs presented the influence of lifestyle on stroke independent of BP. All models were adjusted for age, sex, education level, household income, family history of stroke or heart attack, sedentary behavior, use of antihypertensive drugs, diabetes, respiratory disease, kidney disease, and digestive disease. The proportion of the effect mediated by BP was calculated as NIE/(NDE + NIE). The present study identified an interaction between sex and lifestyle, as well as between sex and BP. Therefore, both pooled and sex-stratified effects were estimated. The bootstrap method (1000 replicates) was used to test the significance of direct and indirect effects.

All statistical analyses were performed using R software (version 4.4.3; The R Foundation for Statistical Computing, Vienna, Austria). A two-tailed *P* < 0.05 was considered statistically significant. Sensitivity analyses were conducted to assess the robustness of our findings. We performed these by: (1) repeating the primary models using categorized BP based on clinical thresholds to evaluate potential non-linearity^[4]^; (2) excluding participants lost to follow-up to assess the impact of attrition; and (3) employing a Fine-Gray competing risk regression model, where non-stroke mortality was treated as a competing event for the stroke outcome.

## Results

Over a median follow-up of 10.76 years (interquartile range: 10.15–11.95), our study included 51929 participants, of whom 30191 (58.14%) were women, with a mean (SD) age of 51.87 (10.28) years. Overall, 16097 participants (31.00%) adhered to four or more healthy lifestyle factors. Individuals with a healthier lifestyle profile were generally younger, more likely to be women, and reported shorter sedentary time, along with a lower prevalence of antihypertensive drug use and diabetes, as well as lower baseline SBP and DBP (***Table 1***).

**Table 1:** Characteristics of participants in the subcohort of the China Kadoorie Biobank study in Suzhou

Characteristics	Number of healthy lifestyle factors^*^						
	Overall	0	1	2	3	4	5
Participants [*n* (%)]	51929 (100.00)	408 (0.79)	3353 (6.46)	12123 (23.34)	19948 (38.41)	14037 (27.03)	2060 (3.97)
Age [years, mean (SD)]	51.87 (10.28)	51.42 (9.32)	51.50 (9.86)	52.97 (10.16)	52.18 (10.35)	51.01 (10.28)	48.98 (10.26)
Women [*n* (%)]	30191 (58.14)	1 (0.25)	42 (1.25)	3775 (31.14)	12391 (62.12)	12115 (86.31)	1867 (90.63)
Highest education level [*n* (%)]							
No formal school	15456 (29.76)	33 (8.09)	362 (10.80)	3054 (25.19)	6465 (32.41)	5072 (36.13)	470 (22.82)
Primary or middle school	31426 (60.52)	313 (76.72)	2552 (76.11)	7815 (64.47)	11760 (58.95)	7702 (54.87)	1284 (62.33)
High school and above	5047 (9.72)	62 (15.20)	439 (13.09)	1254 (10.34)	1723 (8.64)	1263 (9.00)	306 (14.85)
Household income [*n* (%)]							
< 20000 RMB/year	13637 (26.26)	68 (16.67)	727 (21.68)	3312 (27.32)	5477 (27.46)	3728 (26.56)	325 (15.78)
20000–34999 RMB/year	16447 (31.67)	102 (25.00)	878 (26.19)	3618 (29.84)	6420 (32.18)	4740 (33.77)	689 (33.44)
≥ 35000 RMB/year	21845 (42.07)	238 (58.33)	1748 (52.13)	5193 (42.84)	8051 (40.36)	5569 (39.67)	1046 (50.78)
Family history of heart attack or stroke [*n* (%)]	10875 (20.94)	89 (21.81)	738 (22.01)	2564 (21.15)	4249 (21.30)	2812 (20.03)	423 (20.53)
Sedentary behavior > 21 h/week [*n* (%)]	17968 (34.60)	243 (59.56)	1759 (52.46)	5151 (42.49)	6694 (33.56)	3573 (25.45)	548 (26.60)
Usage of antihypertensive drugs [*n* (%)]	8140 (15.68)	94 (23.04)	615 (18.34)	2325 (19.18)	3225 (16.17)	1665 (11.86)	216 (10.49)
Diabetes [*n* (%)]	2655 (5.11)	40 (9.80)	215 (6.41)	803 (6.62)	1002 (5.02)	529 (3.77)	66 (3.20)
Respiratory disease [*n* (%)]	3070 (5.91)	26 (6.37)	231 (6.89)	796 (6.57)	1167 (5.85)	747 (5.32)	103 (5.00)
Kidney disease [*n* (%)]	359 (0.69)	1 (0.25)	16 (0.48)	89 (0.73)	148 (0.74)	90 (0.64)	15 (0.73)
Digestive disease [*n* (%)]	7861 (15.14)	54 (13.24)	492 (14.67)	1916 (15.80)	3136 (15.72)	1961 (13.97)	302 (14.66)
SBP [mmHg, mean (SD)]	132.58 (20.25)	141.13 (20.74)	136.66 (19.57)	134.96 (20.10)	132.42 (20.36)	130.39 (20.11)	126.77 (18.37)
DBP [mmHg, mean (SD)]	78.90 (10.33)	86.57 (11.26)	83.15 (10.99)	80.45 (10.59)	78.50 (10.07)	77.30 (9.78)	76.05 (9.68)
^*^Healthy lifestyle factors: nonsmoking or having stopped for reasons other than illness; nondaily alcohol consumption or daily moderate alcohol consumption (drinking < 25 g of pure alcohol for men and < 15 g for women per day); engaging in an age- (< 50 years, 50–59 years, and ≥ 60 years) and sex-specific median or higher level of physical activity; diet score ≥ 4; and having a body mass index between 18.5 and 27.9 kg/m^2^ and a waist circumference < 90 cm (men)/85 cm (women).Abbreviations: DBP, diastolic blood pressure; SBP, systolic blood pressure; SD, standard deviation.							

A sex-stratified comparison further revealed significant baseline differences. Compared with men, women exhibited a lower cardiovascular risk profile, with significantly lower SBP and DBP. Additionally, women demonstrated a higher prevalence of healthy behaviors, particularly in relation to smoking (defined as never having smoked or having quit for non-illness-related reasons) and moderate alcohol intake (***Supplementary Table 1***). Baseline characteristics stratified by incident stroke status are detailed in ***Supplementary Table 2***. ***Table 2*** presents the results of the correlation analyses among lifestyle factors, SBP, and DBP, indicating a negative correlation between a healthy lifestyle and both SBP and DBP. Harman's single-factor analysis revealed that four factors had eigenvalues greater than 1, with the first factor accounting for 16.11% of the total variance, which was below the critical threshold of 40%. Consequently, common method bias was not a significant concern in the present study.

**Table 2:** Correlations between variables

Variables	1	2	3	4	5	6	7	8	9	10	11	12	13
1. Lifestyle	1												
2. SBP	0.12^**^	1											
3. DBP	0.17^**^	0.72^**^	1										
4. Sex	0.51^**^	0.03^**^	0.11^**^	1									
5. Age	0.06^**^	0.35^**^	0.08^**^	0.03^**^	1								
6. Education	0.10^**^	0.17^**^	0.02^**^	0.33^**^	0.38^**^	1							
7. Family history of heart attack or stroke	0.01^**^	0.09^**^	0.07^**^	0.01^**^	0.13^**^	−0.01	1						
8. Household income	0.02^**^	0.13^**^	−0.01^*^	0.07^**^	0.31^**^	0.28^**^	0.01^**^	1					
9. Sedentary behavior	0.17^**^	−0.01^*^	0.01^**^	0.13^**^	0.01	0.13^**^	0.01	0.05^**^	1				
10. Diabetes	0.05^**^	0.12^**^	0.06^**^	0.00	0.11^**^	0.02^**^	0.03^**^	−0.01^*^	0.02^**^	1			
11. Respiratory disease	0.02^**^	0.01^**^	0.00	0.02^**^	0.10^**^	0.01^*^	0.01^**^	0.04^**^	0.01^**^	0.00	1		
12. Kidney disease	0.00	0.01	0.01	0.03^**^	0.02^**^	−0.01	0.01^*^	0.02^**^	0.01	0.02^**^	0.01^**^	1	
13. Digestive disease	0.01^**^	0.01	−0.01	0.03^**^	0.09^**^	−0.01	0.03^**^	−0.01	0.04^**^	0.04^**^	0.03^**^	0.02^**^	1
The numbers in the columns correspond to the same meanings as the numbers in the rows. For example, column label 1 corresponds to row label 1, which represents "Lifestyle". ^*^P < 0.05 and ^**^P < 0.01.Abbreviations: DBP, diastolic blood pressure; SBP, systolic blood pressure.													

***Table 3*** presents the total, direct, and indirect effects of BP in the association between combined lifestyle factors and stroke. A healthy lifestyle was negatively associated with stroke through both SBP (*β* = −0.0014, 95% CI: −0.0016 to −0.0012) and DBP (*β* = −0.0012, 95% CI: −0.0015 to −0.0009). The proportion of the effect mediated by SBP was 44.70%, while the mediation by DBP accounted for 37.81%. Sex-stratified analyses revealed similar results, with stronger mediating effects in men than in women (SBP: 40.86% *vs.* 39.98%; DBP: 37.06% *vs.* 33.63%). Additionally, we examined the mediating role of BP in the relationship between each of the five individual lifestyle factors and stroke. Diet and alcohol intake factors exhibited significant indirect effects *via* BP (*P* < 0.05), while their direct effects were not statistically significant, suggesting complete mediation (***Supplementary Table 3***).

**Table 3:** Adjusted total, direct, and indirect effects of blood pressure in the association between combined favorable lifestyle factors and stroke

Blood pressure	Overall			Women			Men	
	*β* (95% CI)	*P*-value		*β* (95% CI)	*P*-value		*β* (95% CI)	*P*-value
SBP								
Total effect	−0.0032 (−0.0054–−0.0009)	0.0058		−0.0020 (−0.0051–0.0011)	0.2077		−0.0051 (−0.0085–−0.0018)	0.0023
Direct effect	−0.0017 (−0.004–0.0005)	0.1276		−0.0012 (−0.0043–0.0019)	0.4496		−0.0030 (−0.0064–0.0003)	0.0718
Indirect effect	−0.0014 (−0.0016–−0.0012)	< 0.0001		−0.0008 (−0.0010–−0.0006)	< 0.0001		−0.0021 (−0.0025–−0.0017)	< 0.0001
Proportion mediated (%)	44.70	NA		39.98	NA		40.86	NA
DBP								
Total effect	−0.0032 (−0.0054–−0.0009)	0.0058		−0.0020 (−0.0051–0.0011)	0.2077		−0.0051 (−0.0085–−0.0018)	0.0023
Direct effect	−0.0020 (−0.0042–0.0003)	0.0879		−0.0013 (−0.0044–0.0018)	0.4042		−0.0032 (−0.0066–0.0001)	0.0572
Indirect effect	−0.0012 (−0.0015–−0.0009)	<0.0001		−0.0007 (−0.0009–−0.0004)	<0.0001		−0.0019 (−0.0024–−0.0014)	<0.0001
Proportion mediated (%)	37.81	NA		33.63	NA		37.06	NA
The model was adjusted for age (linear terms), sex (women and men), highest education level (no formal school, primary or middle school, and high school and above), household income (< 20000 RMB/year, 20000–34999 RMB/year, and ≥ 35000 RMB/year), family history of stroke or heart attack (yes, no, and unknown), duration of sedentary behavior (≤ 21 h/week, > 21 h/week), usage of antihypertensive drugs (yes and no), diabetes (yes and no), respiratory disease (yes and no), kidney disease (yes and no), and digestive disease (yes and no).Abbreviations: CI, confidence interval; DBP, diastolic blood pressure; NA, not applicable; SBP, systolic blood pressure.								

We further assessed the mediating role of BP in the association between lifestyle and IS. As shown in ***Table 4***, the inverse association between a healthy lifestyle and IS mirrored that of stroke (*β* = −0.0031, 95% CI: −0.0051 to −0.0010). However, the mediating effects of BP were somewhat reduced (SBP: 33.21%; DBP: 27.24%). The attenuation of these effects was consistent across both women (SBP: 26.07%; DBP: 20.60%) and men (SBP: 36.11%; DBP: 32.64%). Similarly, the BMI-WC, diet, physical activity, and alcohol factors were significantly associated with IS risk through BP-mediated indirect effects (*P* < 0.05), while their direct effects were not statistically significant, indicating complete mediation (***Supplementary Table 4***). These findings remained robust across multiple sensitivity analyses. Our results were largely consistent when: (a) excluding participants lost to follow-up (***Supplementary Tables 5***–***8***); (b) categorizing BP using clinical thresholds (***Supplementary Table 9***); (c) employing a competing risks model that verified the protective effect of a healthy lifestyle (***Supplementary Table 10***); and (d) assessing the overall associations of five favorable individual lifestyle factors and combined favorable lifestyle factors with IS risk (***Supplementary Tables 11*** and ***12***).

**Table 4:** Adjusted total, direct, and indirect effects of blood pressure in the association between combined favorable lifestyle factors and ischemic stroke

Blood pressure	Overall			Women			Men	
	*β* (95% CI)	*P*-value		*β* (95% CI)	*P*-value		*β* (95% CI)	*P*-value
SBP								
Total effect	−0.0031 (−0.0051–−0.0010)	0.0032		−0.0021 (−0.0049–0.0007)	0.1479		−0.0042 (−0.0072–−0.0013)	0.0052
Direct effect	−0.0020 (−0.0041–0.0000)	0.0496		−0.0015 (−0.0044–0.0013)	0.2850		−0.0027 (−0.0057–0.0003)	0.0749
Indirect effect	−0.0010 (−0.0012–−0.0008)	< 0.0001		−0.0005 (−0.0007–−0.0004)	< 0.0001		−0.0015 (−0.0019–−0.0012)	< 0.0001
Proportion mediated (%)	33.21	NA		26.07	NA		36.11	NA
DBP								
Total effect	−0.0031 (−0.0051–−0.0010)	0.0032		−0.0021 (−0.0049–0.0007)	0.1479		−0.0042 (−0.0072–−0.0013)	0.0052
Direct effect	−0.0022 (−0.0043–−0.0002)	0.0331		−0.0017 (−0.0045–0.0012)	0.2518		−0.0028 (−0.0058–0.0001)	0.0622
Indirect effect	−0.0008 (−0.0011–−0.0006)	< 0.0001		−0.0004 (−0.0007–−0.0002)	0.0002		−0.0014 (−0.0018–−0.0009)	< 0.0001
Proportion mediated (%)	27.24	NA		20.60	NA		32.64	NA
The model was adjusted for age (linear terms), sex (women and men), highest education level (no formal school, primary or middle school, and high school and above), household income (< 20000 RMB/year, 20000–34999 RMB/year, and ≥ 35000 RMB/year), family history of stroke or heart attack (yes, no, and unknown), duration of sedentary behavior (≤ 21 h/week, > 21 h/week), usage of antihypertensive drugs (yes and no), diabetes (yes and no), respiratory disease (yes and no), kidney disease (yes and no), and digestive disease (yes and no).Abbreviations: CI, confidence interval; DBP, diastolic blood pressure; NA, not applicable; SBP, systolic blood pressure.								

## Discussion

In this population-based cohort of Chinese adults, we found that the effect of combined lifestyle factors on incident stroke was partly explained through changes in SBP and DBP. Similar results were observed for IS. A sex-specific analysis revealed that the proportion of effect on stroke mediated by BP was higher in men than in women.

Many studies have indicated that adopting a healthy lifestyle reduces stroke risk^[2,7,13,22]^. The Health Professionals Follow-up Study and the Nurses' Health Study examined the effect of five combined lifestyle factors on stroke. Compared with individuals with none of these healthy lifestyle factors, women with all five healthy lifestyle factors had a relative risk of 0.21 for stroke (95% CI: 0.12 to 0.36), while men with all five healthy lifestyle factors had a relative risk of 0.31 (95% CI: 0.19 to 0.53)^[22]^. A study involving over 500000 individuals in China found that adopting a healthy lifestyle reduces the risk of cardiovascular disease and decreases the incidence of IS by 50% (HR = 0.50, 95% CI: 0.40 to 0.64)^[2]^. Moreover, a recent review summarized the epidemiological and laboratory evidence showing that lifestyle interventions play a positive role in the prevention and adjuvant treatment of hypertension^[6]^. However, these studies did not explore the mediating role of BP between combined lifestyle factors and stroke. Consistent with these studies, we found that a healthy lifestyle was negatively associated with stroke. Additionally, after further controlling for all measured confounders, we demonstrated that BP partly mediated the association between lifestyle and stroke. Our findings reinforce the value of lifestyle interventions in preventing hypertension and subsequent stroke.

Our sex-stratified analysis revealed that BP functioned as a significantly stronger mediator in the association between lifestyle and incident stroke in men than in women. To provide a data-driven explanation for this disparity, we further examined the baseline characteristics within our cohort. The analysis demonstrated that, compared with women, men had a significantly higher prevalence of key pro-hypertensive lifestyle factors, including smoking and excessive alcohol consumption. Consistent with this greater burden of adverse behaviors, men also exhibited significantly higher mean SBP and DBP at baseline (SBP: 133.32 [± 18.98] mmHg *vs.* 132.05 [± 21.10] mmHg, *P* < 0.0001; DBP: 80.27 [± 10.22] mmHg *vs.* 77.92 [± 9.94] mmHg, *P* < 0.0001). These findings provide a compelling link to the observed differences in mediation. It is well-established that nicotine and long-term alcohol consumption contribute to elevated BP through mechanisms such as sympathetic nervous system activation and vascular endothelial dysfunction^[23–24]^. Therefore, the higher prevalence of these specific behaviors in men plausibly explains their higher baseline BP levels, which in turn amplifies the importance of BP as a critical mediator in the causal pathway from an unhealthy lifestyle to stroke. While these behavioral patterns provide a direct explanation, underlying pathophysiological mechanisms may also contribute to the sex-specific findings. Furthermore, there are known sex differences in the regulation of BP. For instance, sex hormones such as testosterone and estrogen have been demonstrated to affect numerous pathways related to BP control^[25]^. Compared with men, women exhibit upregulation of angiotensin Ⅱ type 2 receptor (AT2R) expression, which relies on both estrogen and the XX sex chromosome complement^[26]^; and growing evidence supports that AT2R plays a sex-specific role in cardiovascular protection^[27]^. Consequently, sex disparities at any stage of the exposure-mediator-outcome pathway—from lifestyle patterns to hormonal regulation of BP—may serve as potential contributors to the differential mediation effects observed between women and men. In summary, these findings underscore the necessity of sex-specific public health strategies. For men, interventions targeting smoking and alcohol consumption may be particularly effective in mitigating BP-mediated stroke risk.

The present study highlights a key mechanism: SBP mediates over 40% of the protective association between a healthy lifestyle and stroke. This positions SBP not only as a primary risk factor but also as an early, critical indicator for evaluating the efficacy of lifestyle interventions. Further analysis revealed that the effects of physical activity and a healthy BMI-WC were fully mediated by blood pressure in IS, but not in total stroke. This distinction is likely attributed to the inclusion of hemorrhagic stroke, which is directly driven by BP-induced vessel rupture^[28]^, thereby diluting the mediation effect observed in IS^[29]^. Notably, both a healthy diet and moderate alcohol consumption exhibited full mediation for both stroke subtypes, underscoring their broad effect.

These nuanced findings have significant implications for both public health and clinical practice, indicating that population-level BP monitoring may effectively track the success of lifestyle interventions. Clinically, these findings enable healthcare providers to emphasize that lowering BP is the key pathway through which lifestyle changes prevent stroke, making it a critical and measurable goal for patient management.

Several limitations must be acknowledged. First, both lifestyle factors and BP were assessed only at baseline, which may not reflect long-term behaviors or physiological changes during the follow-up period. Although previous studies have shown that most participants maintained stable lifestyle patterns over time^[30]^, the absence of repeated measures restricts our capacity to capture potential changes and weakens causal inference. Future studies are needed to address this limitation by incorporating repeated assessments throughout the follow-up period. Additionally, methods such as dynamic SEM or latent growth modeling may facilitate the elucidation of the evolving relationships between lifestyle, BP, and stroke risk. Second, mediation analysis models assume the absence of exposure-induced mediator-outcome confounding. Unmeasured confounders (*e.g.*, genetic susceptibility) may distort the nature of the association. Nevertheless, adjustment for measured confounders (*e.g.*, a family history of stroke) could help mitigate the potential influence of unmeasured confounders. Third, our study sample was limited to middle-aged and elderly participants, which may restrict the generalizability of our findings to younger populations.

In conclusion, we found that approximately 40% of the protective association between combined lifestyle factors and stroke was mediated by BP. BP may serve as a crucial, early indicator for evaluating the efficacy of lifestyle interventions in reducing stroke risk.

## Additional information

The online version contains supplementary material available at http://www.jbr-pub.org.cn/article/doi/10.7555/JBR.39.20250177?pageType=en.

## References

[1] (2021). Temporal trend and attributable risk factors of stroke burden in China, 1990–2019: An analysis for the Global Burden of Disease Study 2019. Lancet Public Health.

[2] (2017). Adherence to healthy lifestyle and cardiovascular diseases in the Chinese population. J Am Coll Cardiol.

[3] (2018). 2017 ACC/AHA/AAPA/ABC/ACPM/AGS/APhA/ASH/ASPC/NMA/PCNA Guideline for the Prevention, Detection, Evaluation, and Management of High Blood Pressure in Adults: Executive Summary: A Report of the American College of Cardiology/American Heart Association Task Force on Clinical Practice Guidelines. Circulation,.

[4] (2025). Chinese Guidelines for the Prevention and Treatment of Hypertension (2024 revision). J Geriatr Cardiol,.

[5] (2016). Predicting the 10-year risks of atherosclerotic cardiovascular disease in Chinese population: The China-PAR Project (Prediction for ASCVD Risk in China). Circulation.

[6] (2021). Lifestyle interventions for the prevention and treatment of hypertension. Nat Rev Cardiol.

[7] (2018). Genetic risk, incident stroke, and the benefits of adhering to a healthy lifestyle: Cohort study of 306 473 UK Biobank participants. BMJ.

[8] (2016). Blood pressure lowering for prevention of cardiovascular disease and death: A systematic review and meta-analysis. Lancet.

[9] (2022). Development of a model to predict 10-year risk of ischemic and hemorrhagic stroke and ischemic heart disease using the China Kadoorie Biobank. Neurology.

[10] (2011). China Kadoorie Biobank of 0.5 million people: Survey methods, baseline characteristics and long-term follow-up. Int J Epidemiol.

[11] (2013). Physical activity and sedentary leisure time and their associations with BMI, waist circumference, and percentage body fat in 0.5 million adults: The China Kadoorie Biobank study. Am J Clin Nutr.

[12] (2022). The relative validity and reproducibility of food frequency questionnaires in the China Kadoorie Biobank study. Nutrients.

[13] (2023). Association of healthy lifestyle with incident cardiovascular diseases among hypertensive and normotensive Chinese adults. Front Cardiovasc Med.

[14] (2018). Associations of egg consumption with cardiovascular disease in a cohort study of 0.5 million Chinese adults. Heart.

[15] (2016). Fresh fruit consumption and major cardiovascular disease in China. N Engl J Med.

[16] (2016). Dietary Guidelines for Chinese Residents (2016): Comments and comparisons. J Zhejiang Univ Sci B.

[17] (2019). Associations between stressful life events and diabetes: Findings from the China Kadoorie Biobank study of 500000 adults. J Diabetes Investig.

[18] (2014). Diagnosis and classification of diabetes mellitus. Diabetes Care.

[19] (2024). Comprehensive nursing model for diabetic foot ulcers: A strategy to improve prognosis and quality of life. Medicine (Baltimore).

[20] (2000). Marginal structural models and causal inference in epidemiology. Epidemiology.

[21] (2013). Introduction to mediation analysis with structural equation modeling. Shanghai Arch Psychiatry.

[22] (2008). Primary prevention of stroke by healthy lifestyle. Circulation.

[23] (2019). Cardiovascular injury induced by tobacco products: Assessment of risk factors and biomarkers of harm. A Tobacco Centers of Regulatory Science compilation. Am J Physiol Heart Circ Physiol.

[24] (2011). Chronic ethanol ingestion induces aortic inflammation/oxidative endothelial injury and hypertension in rats. Hum Exp Toxicol.

[25] (2022). Blood pressure and stroke: A review of sex- and ethnic/racial-specific attributes to the epidemiology, pathophysiology, and management of raised blood pressure. Stroke.

[26] (2015). Angiotensin Ⅱ type 2 receptor- and acetylcholine-mediated relaxation: Essential contribution of female sex hormones and chromosomes. Hypertension.

[27] (2016). Sex differences in hypertension: Recent advances. Hypertension.

[28] (2024). Epidemiology, pathophysiology, and current treatment strategies in stroke. Cardiol Cardiovasc Med.

[29] (2021). Association of lifestyle change with incident stroke and its subtypes. Chin J Dis Control Prev.

[30] (2021). Lifestyle, cardiometabolic disease, and multimorbidity in a prospective Chinese study. Eur Heart J.

