# Supplementary material for: The mediating effect of blood pressure between healthy lifestyles and stroke: Results from the China Kadoorie Biobank study
Source: J Biomed Res. 2025 Aug 29;40(1):23–31. doi: 10.7555/JBR.39.20250177 (PMC12799335; doi:10.7555/JBR.39.20250177)
Supplement: Supplementary file 1 — The online version contains supplementary material available at http://www.jbr-pub.org.cn/article/doi/10.7555/JBR.39.20250177?pageType=en. [file jbr-40-1-23-S1.pdf]

Available online at www.jbr-pub.org.cn

Open Access at PubMed Central

Journal of Biomedical Research, 2026 40(1): 1–12

Supplementary Data

JBR

The mediating effect of blood pressure between healthy lifestyles and stroke: Results from the China Kadoorie Biobank study

Zidong Wang[1,△], Jiaxi Zhou[1,△], Xikang Fan[2], Jian Su[1,2], Houyue Geng[1], Xun Wu[2], Yujie Hua[3], Hongfu Ren[4], Jun Lyu[5,6,7], Pei Pei[6], Canqing Yu[5,6,7], Dianjianyi Sun[5,6,7], Yan Lu[3], Jinyi Zhou[1,2], Ran Tao[1,2,✉]

[1]*School of Public Health, Nanjing Medical University, Nanjing, Jiangsu 211166, China;*
[2]*Department of Noncommunicable Chronic Disease Control and Prevention, Jiangsu Provincial Center for Disease Control and Prevention, Nanjing, Jiangsu 210009, China;*
[3]*Department of Noncommunicable Chronic Disease Control and Prevention, Suzhou City Center for Disease Control and Prevention, Suzhou, Jiangsu 215004, China;*
[4]*Department of Noncommunicable Chronic Disease Control and Prevention, Wuzhong District of Suzhou City Center for Disease Control and Prevention, Suzhou, Jiangsu 215100, China;*
[5]*Department of Epidemiology and Biostatistics, School of Public Health, Peking University, Beijing 100191, China;*
[6]*Public Health and Epidemic Preparedness and Response Center, Peking University, Beijing 100191, China;*
[7]*Key Laboratory of Epidemiology of Major Diseases, Ministry of Education (Peking University), Beijing 100191, China.*

Supplementary Table 1 **Baseline characteristics of participants stratified by sex**

Characteristics	Overall	Female	Male	*P*-value
Participants [*n* (%)]	51 929 (100.00)	30 191 (58.1)	21 738 (41.9)	< 0.000 1
Age [years, mean (SD)]	51.64 (10.28)	51.61 (10.30)	52.23 (10.25)	< 0.000 1
Highest education level [*n* (%)]				< 0.000 1
No formal school	15 456 (29.76)	13 165 (43.61)	2 291 (10.54)	
Primary or middle school	31 426 (60.52)	14 894 (49.33)	16 532 (76.05)	
High school and above	5 047 (9.72)	2 132 (7.06)	2 915 (13.41)	
Household income [*n* (%)]				< 0.000 1
< 20 000 RMB/year	13 637 (26.26)	8 466 (28.04)	5 171 (23.79)	
20 000–34 999 RMB/year	16 447 (31.67)	9 999 (33.12)	6 448 (29.66)	
≥ 35 000 RMB/year	21 845 (42.07)	11 726 (38.84)	10 119 (46.55)	

△These authors contributed equally to this work.

✉Corresponding author: Ran Tao, School of Public Health, Nanjing Medical University, 101 Longmian Avenue, Nanjing, Jiangsu 211166, China. E-mail: trltjy@163.com.

Received: 21 April 2025; Revised: 12 August 2025; Accepted: 18 August 2025; Published online: 29 August 2025

CLC number: R544.1, Document code: A

The authors reported no conflict of interests.

This is an open access article under the Creative Commons Attribution (CC BY 4.0) license, which permits others to distribute, remix, adapt and build upon this work, for commercial use, provided the original work is properly cited.

© 2026 by Journal of Biomedical Research.

https://doi.org/10.7555/JBR.39.20250177

Supplementary Table 1 **Baseline characteristics of participants stratified by sex** (continued)

Characteristics	Overall	Women	Men	*P*-value
Family history of heart attack or stroke [*n* (%)]	10 875 (20.94)	6 172 (20.44)	4 703 (21.63)	0.004 1
Sedentary behavior > 21 h/week [*n* (%)]	17 968 (34.60)	8 820 (29.21)	9 148 (42.08)	< 0.000 1
Usage of antihypertensive drugs [*n* (%)]	8 140 (15.68)	4 781 (18.54)	3 359 (15.45)	0.240 4
Diabetes [*n* (%)]	2 655 (5.11)	1 544 (5.11)	1 111 (5.11)	1.000 0
Respiratory disease [*n* (%)]	3 070 (5.91)	1 657 (5.49)	1 413 (6.50)	< 0.000 1
Kidney disease [*n* (%)]	359 (0.69)	268 (0.88)	91 (0.42)	< 0.000 1
Digestive disease [*n* (%)]	7 861 (15.14)	4 821 (15.97)	3 040 (13.98)	< 0.000 1
SBP [mmHg, mean (SD)]	132.58 (20.25)	132.05 (21.10)	133.32 (18.98)	< 0.000 1
DBP [mmHg, mean (SD)]	78.90 (10.33)	77.92 (9.94)	80.27 (10.22)	< 0.000 1
Favorable BWI-WC [*n* (%)][a]	37 898 (72.98)	21 383 (70.83)	16 515 (75.97)	< 0.000 1
Favorable diet [*n* (%)][b]	9 521 (18.33)	6 046 (20.03)	3 475 (15.99)	< 0.000 1
Favorable MET [*n* (%)][c]	25 896 (49.87)	15 033 (49.79)	10 863 (49.97)	0.693 2
Favorable alcohol intake [*n* (%)][d]	46 894 (90.30)	30 158 (99.89)	16 736 (76.99)	< 0.000 1
Favorable smoke [*n* (%)][e]	33 682 (64.86)	29 940 (99.17)	3 742 (17.21)	< 0.000 1

[a]BMI between 18.5 and 27.9 kg/m^2 and a WC < 90 cm (men)/85 cm (women).
[b]Diet score ≥ 4.
[c]Engaging in an age- (< 50 years, 50–59 years, and ≥ 60 years) and sex-specific median or higher level of physical activity.
[d]Nondaily alcohol consumption or daily moderate alcohol consumption (drinking < 25 g of pure alcohol for men and < 15 g for women per day).
[e]Nonsmoking or having stopped for reasons other than illness.
Abbreviations: BMI, body mass index; DBP, diastolic blood pressure; SBP, systolic blood pressure; SD, standard deviation; WC, waist circumference.

Supplementary Table 2 **Baseline characteristics of participants stratified by incident stroke**

Characteristics	Overall	Stroke	Non-stroke	*P*-value
Participants (*n*)	51 929	2 811	49 118	
Age [years, mean (SD)]	51.87 (10.28)	61.53 (9.05)	51.31 (10.07)	< 0.000 1
Women	30 191 (58.14)	1 501 (53.40)	28 690 (58.41)	< 0.000 1
Highest education level [*n* (%)]				< 0.000 1
No formal school	15 456 (29.76)	1 366 (48.60)	14 090 (28.69)	
Primary or middle school	31 426 (60.52)	1 291 (45.93)	30 135 (61.35)	
High school and above	5 047 (9.72)	154 (5.48)	4 893 (9.96)	
Household income [*n* (%)]				< 0.000 1
< 20 000 RMB/year	13 637 (26.26)	1 300 (46.25)	12 337 (25.12)	
20 000–34 999 RMB/year	16 447 (31.67)	676 (24.05)	15 771 (32.11)	
≥ 35 000 RMB/year	21 845 (42.07)	835 (29.70)	21 010 (42.47)	
Family history of heart attack or stroke [*n* (%)]	10 875 (20.94)	747 (26.57)	10 128 (20.62)	< 0.000 1
Sedentary behavior > 21 h/week [*n* (%)]	17 968 (34.60)	21.82 (16.85)	21.18 (14.05)	0.014 6
Usage of antihypertensive drugs [*n* (%)]	8 140 (15.68)	1 002 (35.65)	7 138 (14.53)	< 0.000 1
Diabetes [*n* (%)]	2 655 (5.11)	324 (11.53)	2 331 (4.75)	< 0.000 1
Respiratory disease [*n* (%)]	3 070 (5.91)	240 (8.54)	2 830 (5.76)	< 0.000 1
Kidney disease [*n* (%)]	359 (0.69)	33 (1.17)	326 (0.66)	0.002 2
Digestive disease [*n* (%)]	7 861 (15.14)	539 (19.17)	7 322 (14.91)	< 0.000 1
SBP (mmHg, mean [SD])	132.58 (20.25)	145.92 (24.05)	131.82 (19.74)	< 0.000 1
DBP (mmHg, mean [SD])	78.90 (10.33)	82.22 (11.67)	78.71 (10.22)	< 0.000 1

Abbreviations: DBP, diastolic blood pressure; SBP, systolic blood pressure; SD, standard deviation.

Supplementary Table 3 **Adjusted direct and indirect associations of five favorable individual lifestyle factors with stroke mediated *via* blood pressure**

Blood pressure	Favorable BMI-WC[a]		Favorable diet[b]		Favorable physical activity[c]		Favorable alcohol[d]		Favorable smoke[e]	
	β (95% CI)	*P*-value	β (95% CI)	*P*-value	β (95% CI)	*P*-value	β (95% CI)	*P*-value	β (95% CI)	*P*-value
SBP										
Total effect	0.001 2 (−0.003 1–0.005 6)	0.581 4	−0.004 0 (−0.009 1–0.001 0)	0.116 1	−0.005 (−0.008 8–−0.001 1)	0.011 9	−0.002 6 (−0.009 5–0.004 3)	0.461 1	−0.008 1 (−0.015 6–−0.000 6)	0.033 1
Direct effect	0.004 7 (0.000 4–0.009 1)	0.035 5	−0.002 5 (−0.007 5–0.002 5)	0.325 0	−0.005 2 (−0.009–−0.001 3)	0.008 5	0.000 9 (−0.006 1–0.007 8)	0.806 8	−0.009 1 (−0.016 6–−0.001 7)	0.016 5
Indirect effect	−0.003 5 (−0.004 0–−0.003 0)	< 0.000 1	−0.001 5 (−0.001 9–−0.001 1)	< 0.000 1	0.000 2 (0.000 0–0.000 5)	0.090 0	−0.003 5 (−0.004 1–−0.002 8)	< 0.000 1	0.001 0 (0.000 5–0.001 5)	0.000 1
Proportion mediated (%)	−286.85	NA	37.46	NA	−4.5	NA	133.17	NA	−12.28	NA
DBP										
Total effect	0.001 2 (−0.003 1–0.005 6)	0.581 4	−0.004 (−0.009 1–0.001)	0.116 1	−0.005 (−0.008 8–−0.001 1)	0.011 9	−0.002 6 (−0.009 5–0.004 3)	0.461 1	−0.008 1 (−0.015 6–−0.000 6)	0.033 1
Direct effect	0.004 1 (−0.000 3–0.008 5)	0.067 9	−0.003 3 (−0.008 4–0.001 7)	0.192 1	−0.004 7 (−0.008 5–−0.000 8)	0.018 1	0.000 1 (−0.006 8–0.007 1)	0.968 4	−0.008 7 (−0.016 2–−0.001 3)	0.022 1
Indirect effect	−0.002 9 (−0.003 5–−0.002 3)	< 0.000 1	−0.000 7 (−0.000 9–−0.000 4)	< 0.000 1	−0.000 3 (−0.000 5–−0.000 1)	0.000 6	−0.002 7 (−0.003 4–−0.002 1)	< 0.000 1	0.000 6 (0.000 3–0.000 9)	0.000 5
Proportion mediated (%)	−233.74	NA	17.02	NA	6.1	NA	105.39	NA	−7.35	NA

The model was adjusted for age (linear terms), sex (women and men), highest education level (no formal school, primary or middle school, and high school and above), household income (< 20 000 RMB/year, 20 000–34 999 RMB/year, and ≥ 35 000 RMB/year), family history of stroke or heart attack (yes, no, and unknown), duration of sedentary behavior (≤ 21 h/week, > 21 h/week), usage of antihypertensive drugs (yes and no), diabetes (yes and no), respiratory disease (yes and no), kidney disease (yes and no), and digestive disease (yes and no).

[a]BMI between 18.5 and 27.9 kg/m^2 and a WC < 90 cm (men)/85 cm (women).

[b]Diet score ≥ 4.

[c]Engaging in an age- (< 50 years, 50–59 years, and ≥ 60 years) and sex-specific median or higher level of physical activity.

[d]Nondaily alcohol consumption or daily moderate alcohol consumption (drinking < 25 g of pure alcohol for men and < 15 g for women per day).

[e]Nonsmoking or having stopped for reasons other than illness.

Abbreviations: CI, confidence interval; DBP, diastolic blood pressure; NA, not applicable; SBP, systolic blood pressure.

Supplementary Table 4 **Adjusted direct and indirect associations of five favorable individual lifestyle factors with ischemic stroke mediated *via* blood pressure**

Blood pressure	Favorable BMI-WC[a]		Favorable diet[b]		Favorable physical activity[c]		Favorable alcohol[d]		Favorable smoke[e]	
	β (95% CI)	*P*-value	β (95% CI)	*P*-value	β (95% CI)	*P*-value	β (95% CI)	*P*-value	β (95% CI)	*P*-value
SBP										
Total effect	−0.001 7 (−0.005 6–0.002 2)	0.402 0	−0.003 6 (−0.008 2–0.000 9)	0.118 2	−0.001 7 (−0.005 6–0.002 2)	0.402 0	−0.001 1 (−0.007 3–0.005 2)	0.734 7	−0.006 5 (−0.013 3–0.000 3)	0.059 3
Direct effect	0.000 8 (−0.003 1–0.004 8)	0.683 5	−0.002 5 (−0.007 1–0.002)	0.273 8	0.000 3 (−0.003 7–0.004 2)	0.886 8	0.001 4 (−0.004 8–0.007 7)	0.656 8	−0.007 2 (−0.014–−0.000 5)	0.036 0
Indirect effect	−0.002 5 (−0.003–−0.002)	< 0.000 1	−0.001 1 (−0.001 4–−0.000 8)	< 0.000 1	−0.002 (−0.002 5–−0.001 4)	< 0.000 1	−0.002 5 (−0.003–−0.002)	< 0.000 1	0.000 7 (0.000 3–0.001 1)	0.000 2
Proportion mediated (%)	148.84	NA	30.00	NA	117.12	NA	231.21	NA	−11.06	NA
DBP										
Total effect	−0.001 7 (−0.005 6–0.002 2)	0.402 0	−0.003 6 (−0.008 2–0.000 9)	0.118 2	−0.003 5 (−0.007–0)	0.050 5	−0.001 1 (−0.007 3–0.005 2)	0.734 7	−0.006 5 (−0.013 3–0.000 3)	0.059 3
Direct effect	0.000 3 (−0.003 7–0.004 2)	0.886 8	−0.003 1 (−0.007 7–0.001 4)	0.175 6	−0.003 3 (−0.006 8–0.000 2)	0.066 2	0.000 9 (−0.005 4–0.007 1)	0.789 6	−0.006 9 (−0.013 7–−0.000 2)	0.044 6
Indirect effect	−0.002 (−0.002 5–−0.001 4)	< 0.000 1	−0.000 5 (−0.000 7–−0.000 3)	< 0.000 1	−0.000 2 (−0.000 3–−0.000 1)	0.001 0	−0.001 9 (−0.002 5–−0.001 4)	< 0.000 1	0.000 4 (0.000 2–0.000 7)	0.000 9
Proportion mediated (%)	117.12	NA	13.32	NA	6.11	NA	178.96	NA	−6.47	NA

The model was adjusted for age (linear terms), sex (women and men), highest education level (no formal school, primary or middle school, and high school and above), household income (< 20 000 RMB/year, 20 000–34 999 RMB/year, and ≥ 35 000 RMB/year), family history of stroke or heart attack (yes, no, and unknown), duration of sedentary behavior (≤ 21 h/week, > 21 h/week), usage of antihypertensive drugs (yes and no), diabetes (yes and no), respiratory disease (yes and no), kidney disease (yes and no), and digestive disease (yes and no).

[a]BMI between 18.5 and 27.9 kg/m^2 and a WC < 90 cm (men)/85 cm (women).

[b]Diet score ≥ 4.

[c]Engaging in an age- (< 50 years, 50–59 years, and ≥ 60 years) and sex-specific median or higher level of physical activity.

[d]Nondaily alcohol consumption or daily moderate alcohol consumption (drinking < 25 g of pure alcohol for men and < 15 g for women per day).

[e]Nonsmoking or having stopped for reasons other than illness.

Abbreviations: BMI, body mass index; CI, confidence interval; DBP, diastolic blood pressure; NA, not applicable; SBP, systolic blood pressure; WC, waist circumference.

Supplementary Table 5 **Adjusted direct and indirect associations of combined favorable lifestyle factors with stroke mediated *via* blood pressure (excluding participants lost-follow-up)**

Blood pressure	Overall		Women		Men	
	β (95% CI)	*P*-value	*β* (95% CI)	*P*-value	*β* (95% CI)	*P*-value
SBP						
Total effect	−0.003 1 (−0.005 3–−0.000 8)	0.007 6	−0.002 (−0.005 1–0.001 2)	0.216 0	−0.005 (−0.008 3–−0.001 7)	0.003 3
Direct effect	−0.001 7 (−0.003 9–0.000 6)	0.153 0	−0.001 2 (−0.004 3–0.002)	0.464 0	−0.002 9 (−0.006 2–0.000 5)	0.091 4
Indirect effect	−0.001 4 (−0.001 7–−0.001 2)	< 0.000 1	−8e−04 (−0.001–−0.000 6)	< 0.000 1	−0.002 1 (−0.002 6–−0.001 7)	< 0.000 1
Proportion mediated (%)	46.37	NA	40.82	NA	42.52	NA
DBP						
Total effect	−0.003 1 (−0.005 3–−0.000 8)	0.007 6	−0.002 (−0.005 1–0.001 2)	0.216 0	−0.005 (−0.008 3–−0.001 7)	0.003 3
Direct effect	−0.001 9 (−0.004 1–0.000 4)	0.107 7	−0.001 3 (−0.004 4–0.001 8)	0.417 3	−0.003 1 (−0.006 4–0.000 3)	0.074 2
Indirect effect	−0.001 2 (−0.001 5–−0.000 9)	< 0.000 1	−0.000 7 (−0.000 9–−0.000 4)	< 0.000 1	−0.001 9 (−0.002 5–−0.001 4)	< 0.000 1
Proportion mediated (%)	39.37	NA	34.29	NA	38.74	NA

The model was adjusted for age (linear terms), sex (women and men), highest education level (no formal school, primary or middle school, and high school and above), household income (< 20 000 RMB/year, 20 000–34 999 RMB/year, and ≥ 35 000 RMB/year), family history of stroke or heart attack (yes, no, and unknown), duration of sedentary behavior (≤ 21 h/week, > 21 h/week), usage of antihypertensive drugs (yes and no), diabetes (yes and no), respiratory disease (yes and no), kidney disease (yes and no), and digestive disease (yes and no).
Abbreviations: CI: confidence interval; DBP, diastolic blood pressure; NA, not applicable; SBP, systolic blood pressure.

Supplementary Table 6 **Adjusted direct and indirect associations of combined favorable lifestyle factors with ischemic stroke mediated *via* blood pressure (excluding participants lost-follow-up)**

Blood pressure	Overall		Women		Men	
	β (95% CI)	*P*-value	β (95% CI)	*P*-value	β (95% CI)	*P*-value
SBP						
Total effect	−0.003 (−0.005–−0.000 9)	0.004 2	−0.002 1 (−0.005–0.000 8)	0.153 5	−0.004 1 (−0.007 1–−0.001 1)	0.007 0
Direct effect	−0.002 (−0.004–0.000 1)	0.060 0	−0.001 5 (−0.004 4–0.001 3)	0.294 2	−0.002 6 (−0.005 5–0.000 4)	0.093 1
Indirect effect	−0.001 (−0.001 2–−0.000 8)	< 0.000 1	−0.000 6 (−0.000 7–−0.000 4)	< 0.000 1	−0.001 5 (−0.001 9–−0.001 2)	< 0.000 1
Proportion mediated (%)	34.23	NA	26.47	NA	37.56	NA
DBP						
Total effect	−0.003 (−0.005 0–−0.000 9)	0.004 2	−0.002 1 (−0.005–0.000 8)	0.153 5	−0.004 1 (−0.007 1–−0.001 1)	0.007 0
Direct effect	−0.002 2 (−0.004 2–−0.000 1)	0.040 7	−0.001 7 (−0.004 5–0.001 2)	0.260 0	−0.002 7 (−0.005 7–0.000 3)	0.078 4
Indirect effect	−0.000 8 (−0.001 1–−0.000 6)	< 0.000 1	−0.000 4 (−0.000 7–−0.000 2)	0.000 2	−0.001 4 (−0.001 8–−0.000 9)	< 0.000 1
Proportion mediated (%)	28.18	NA	20.88	NA	34.11	NA

The model was adjusted for age (linear terms), sex (women and men), highest education level (no formal school, primary or middle school, and high school and above), household income (< 20 000 RMB/year, 20 000–34 999 RMB/year, and ≥ 35 000 RMB/year), family history of stroke or heart attack (yes, no, and unknown), duration of sedentary behavior (≤ 21 h/week, > 21 h/week), usage of antihypertensive drugs (yes and no), diabetes (yes and no), respiratory disease (yes and no), kidney disease (yes and no), and digestive disease (yes and no).
Abbreviations: CI, confidence interval; DBP, diastolic blood pressure; NA, not applicable; SBP, systolic blood pressure.

Supplementary Table 7 **Adjusted direct and indirect associations of five favorable individual lifestyle factors with stroke mediated *via* blood pressure (rxcluding participants lost-follow-up)**

Blood pressure	Favorable BMI-WC[a]		Favorable diet[b]		Favorable physical activity[c]		Favorable alcohol[d]		Favorable smoke[e]	
	β (95% CI)	*P*-value	β (95% CI)	*P*-value	β (95% CI)	*P*-value	β (95% CI)	*P*-value	β (95% CI)	*P*-value
SBP										
Total effect	0.001 1 (−0.003 3–0.005 5)	0.619 9	−0.003 5 (−0.008 6–0.001 6)	0.176 2	−0.005 2 (−0.009 1–−0.001 3)	0.009 4	−0.002 4 (−0.009 3–0.004 6)	0.502 7	−0.007 6 (−0.015 1–0)	0.048 8
Direct effect	0.004 7 (0.000 3–0.009 1)	0.037 9	−0.002 (−0.007 1–0.003 1)	0.444 6	−0.005 4 (−0.009 3–−0.001 5)	0.006 7	0.001 1 (−0.005 8–0.008 1)	0.754 8	−0.008 6 (−0.016 2–−0.001 1)	0.024 7
Indirect effect	−0.003 5 (−0.004 1–−0.003)	< 0.000 1	−0.001 5 (−0.001 9–−0.001 1)	< 0.000 1	0.000 4 (< 0.000 1–0.000 5)	0.103 9	−0.003 5 (−0.004 1–−0.002 8)	< 0.000 1	0.001 (0.000 5–0.001 6)	0.000 1
Proportion mediated (%)	−319.78	NA	43.56	NA	−4.19	NA	146.59	NA	−13.74	NA
DBP										
Total effect	0.001 1 (−0.003 3–0.005 5)	0.619 9	−0.003 5 (−0.008 6–0.001 6)	0.176 2	−0.005 2 (−0.009 1–−0.001 3)	0.009 4	−0.002 4 (−0.009 3–0.004 6)	0.502 7	−0.007 6 (−0.015 1–0)	0.048 8
Direct effect	0.004 (−0.000 4–0.008 4)	0.076 3	−0.002 8 (−0.007 9–0.002 3)	0.278 8	−0.004 8 (−0.008 7–−0.001)	0.014 6	0.000 4 (−0.006 6–0.007 4)	0.912 9	−0.008 2 (−0.015 7–−0.000 6)	0.033 3
Indirect effect	−0.002 9 (−0.003 5–−0.002 3)	< 0.000 1	−0.000 7 (−0.001–−0.000 4)	< 0.000 1	−0.000 3 (−0.000 5–−0.000 4)	0.000 5	−0.002 8 (−0.003 4–−0.002 1)	< 0.000 1	0.000 4 (0.000 3–0.000 9)	0.000 5
Proportion mediated (%)	0.001 1	NA	19.96	NA	6.07	NA	116.37	NA	−7.94	NA

The model was adjusted for age (linear terms), sex (women and men), highest education level (no formal school, primary or middle school, and high school and above), household income (< 20 000 RMB/year, 20 000–34 999 RMB/year, and ⩾ 35 000 RMB/year), family history of stroke or heart attack (yes, no, and unknown), duration of sedentary behavior (⩽ 21 h/week, > 21 h/week), usage of antihypertensive drugs (yes and no), diabetes (yes and no), respiratory disease (yes and no), kidney disease (yes and no), and digestive disease (yes and no).

[a]BMI between 18.5 and 27.9 kg/m^2 and a WC < 90 cm (men)/85 cm (women).

[b]Diet score ⩾ 4.

[c]Engaging in an age- (< 50 years, 50–59 years, and ⩾ 60 years) and sex-specific median or higher level of physical activity.

[d]Nondaily alcohol consumption or daily moderate alcohol consumption (drinking < 25 g of pure alcohol for men and < 15 g for women per day).

[e]Nonsmoking or having stopped for reasons other than illness.

Abbreviations: BMI, body mass index; CI: confidence interval; DBP, diastolic blood pressure; NA, not applicable; SBP, systolic blood pressure; WC, waist circumference.

Supplementary Table 8 **Adjusted direct and indirect associations of five favorable individual lifestyle factors with ischemic stroke mediated *via* blood pressure (excluding participants lost-follow-up)**

Blood pressure	Favorable BMI-WC[a]		Favorable diet[b]		Favorable physical activity[c]		Favorable alcohol[d]		Favorable smoke[e]	
	β (95% CI)	*P*-value	β (95% CI)	*P*-value	β (95% CI)	*P*-value	β (95% CI)	*P*-value	β (95% CI)	*P*-value
SBP										
Total effect	−0.001 8 (−0.005 8–0.002 2)	0.374 9	−0.003 2 (−0.007 8–0.001 4)	0.171 1	−0.003 6 (−0.007 2–−0.000 1)	0.042 5	−0.000 9 (−0.007 2–0.005 4)	0.777 2	−0.006 1 (−0.012 9–0.000 8)	0.081 1
Direct effect	0.000 4 (−0.003 2–0.004 7)	0.717 9	−0.002 1 (−0.006 7–0.002 5)	0.368 0	−0.003 8 (−0.007 3–−0.000 3)	0.034 2	0.001 6 (−0.004 7–0.007 9)	0.616 4	−0.006 8 (−0.013 6–0)	0.049 7
Indirect effect	−0.002 5 (−0.003–−0.002 1)	< 0.000 1	−0.001 1 (−0.001 4–−0.000 8)	< 0.000 1	0.000 2 (0–0.000 3)	0.105 1	−0.002 5 (−0.003–−0.002)	< 0.000 1	0.000 8 (0.000 4–0.001 1)	0.000 1
Proportion mediated (%)	140.88	NA	34.27	NA	−4.27	NA	277.19	NA	−12.37	NA
DBP										
Total effect	−0.001 8 (−0.005 8–0.002 2)	0.374 9	−0.003 2 (−0.007 8–0.001 4)	0.171 1	−0.003 6 (−0.007 2–−0.000 1)	0.042 5	−0.000 9 (−0.007 2–0.005 4)	0.777 2	−0.006 1 (−0.012 9–0.000 8)	0.081 1
Direct effect	0.000 2 (−0.003 8–0.004 2)	0.925 3	−0.002 7 (−0.007 3–0.001 9)	0.246 4	−0.003 4 (−0.006 9–0.000 1)	0.056 5	0.001 (−0.005 3–0.007 4)	0.745 2	−0.006 5 (−0.013 3–0.000 3)	0.061 9
Indirect effect	−0.002 (−0.002 5–−0.001 4)	< 0.000 1	−0.000 5 (−0.000 7–−0.000 3)	< 0.000 1	−0.000 2 (−0.000 3–−0.000 1)	0.000 8	−0.002 (−0.002 5–−0.001 4)	< 0.000 1	0.000 4 (0.004–0.000 7)	0.000 9
Proportion mediated (%)	110.66	NA	15.33	NA	6.04	NA	215.19	NA	−6.98	NA

The model was adjusted for age (linear terms), sex (women and men), highest education level (no formal school, primary or middle school, and high school and above), household income (< 20 000 RMB/year, 20 000–34 999 RMB/year, and ≥ 35 000 RMB/year), family history of stroke or heart attack (yes, no, and unknown), duration of sedentary behavior (≤ 21 h/week, > 21 h/week), usage of antihypertensive drugs (yes and no), diabetes (yes and no), respiratory disease (yes and no), kidney disease (yes and no), and digestive disease (yes and no).

[a]BMI between 18.5 and 27.9 kg/m^2 and a WC < 90 cm (men)/85 cm (women).

[b]Diet score ≥ 4.

[c]Engaging in an age- (< 50 years, 50–59 years, and ≥ 60 years) and sex-specific median or higher level of physical activity.

[d]Nondaily alcohol consumption or daily moderate alcohol consumption (drinking < 25 g of pure alcohol for men and < 15 g for women per day).

[e]Nonsmoking or having stopped for reasons other than illness.

Abbreviations: BMI, body mass index; CI: confidence interval; DBP, diastolic blood pressure; NA, not applicable; SBP, systolic blood pressure; WC, waist circumference.

Supplementary Table 9 **Adjusted mediation effects of combined favorable lifestyle factors on stroke and ischemic stroke *via* blood pressure categorized by clinical thresholds**

Blood pressure	Overall		Women		Men	
	β (95% CI)	*P*-value	β (95% CI)	*P*-value	β (95% CI)	*P*-value
Stroke						
Total effect	−0.003 2 (−0.005 4–−0.000 9)	0.005 9	−0.002 (−0.005 1–0.001 1)	0.209 4	−0.005 1 (−0.008 5–−0.001 8)	0.002 9
Direct effect	−0.002 2 (−0.004 4–0.000 1)	0.058 4	−0.001 4 (−0.004 5–0.001 7)	0.371 7	−0.003 7 (−0.007 1–−0.000 3)	0.031 9
Indirect effect	−0.001 0 (−0.001 2–−0.000 7)	< 0.000 1	−0.000 6 (−0.000 8–−0.000 3)	< 0.000 1	−0.001 4 (−0.001 8–−0.001)	< 0.000 1
Proportion mediated (%)	30.97	NA	28.71	NA	27.73	NA
Ischemic stroke						
Total effect	−0.003 1 (−0.005 1–−0.001)	0.003 4	−0.002 1 (−0.005–0.000 8)	0.151 3	−0.004 2 (−0.007 2–−0.001 2)	0.006 2
Direct effect	−0.002 4 (−0.004 4–−0.000 3)	0.023 1	−0.001 7 (−0.004 6–0.001 2)	0.241 4	−0.003 2 (−0.006 3–−0.000 2)	0.036 2
Indirect effect	−0.000 7 (−0.000 9–−0.000 5)	< 0.000 1	−0.000 4 (−0.000 6–−0.000 2)	0.000 2	−0.001 (−0.001 3–−0.000 6)	< 0.000 1
Proportion mediated (%)	22.16	NA	18.19	NA	23.11	NA

The model was adjusted for age (linear terms), sex (women and men), highest education level (no formal school, primary or middle school, and high school and above), household income (< 20 000 RMB/year, 20 000–34 999 RMB/year, and ≥ 35 000 RMB/year), family history of stroke or heart attack (yes, no, and unknown), duration of sedentary behavior (≤ 21 h/week, > 21 h/week), usage of antihypertensive drugs (yes and no), diabetes (yes and no), respiratory disease (yes and no), kidney disease (yes and no), and digestive disease (yes and no). Blood pressure clinical thresholds: normal blood pressure, SBP < 120 mmHg and DBP < 80 mmHg; high-normal blood pressure, 120 ≤ SBP < 140 mmHg or 80 ≤ DBP < 90 mmHg; hypertension grade 1, 140 ≤ SBP < 160 mmHg or 90 ≤ DBP < 100 mmHg; hypertension grade 2, 160 ≤ SBP < 180 mmHg or 100 ≤ DBP < 110 mmHg; hypertension grade 3, SBP ≥ 180 mmHg or DBP ≥ 110 mmHg.
Abbreviations: DBP, diastolic blood pressure; NA, not applicable; SBP, systolic blood pressure.

Supplementary Table 10 **Results of Fine-Gray competing risks model: associations between lifestyle factors and stroke and ischemic stroke risk**

	Overall	*P*-value	Men	*P*-value	Women	*P*-value
Stroke						
Basic model	0.87 (0.84–0.91)	< 0.000 1	0.87 (0.83–0.93)	< 0.000 1	0.88 (0.82–0.94)	< 0.000 1
Full model	0.91 (0.87–0.95)	< 0.000 1	0.90 (0.85–0.95)	0.000 2	0.93 (0.87–0.99)	0.028 0
Ischemic stroke						
Basic model	0.86 (0.82–0.91)	< 0.000 1	0.87 (0.82–0.93)	< 0.000 1	0.86 (0.80–0.92)	< 0.000 1
Full model	0.90 (0.86–0.95)	< 0.000 1	0.89 (0.84–0.95)	0.000 6	0.92 (0.86–0.99)	0.023 0

Basic model was adjusted for age (linear terms), sex (women and men). The full model was further adjusted for highest education level (no formal school, primary or middle school, and high school and above), household income (< 20 000 RMB/year, 20 000–34 999 RMB/year, and ⩾ 35 000 RMB/year), family history of stroke or heart attack (yes, no, and unknown), duration of sedentary behavior (⩽ 21 h/week, > 21 h/week), usage of antihypertensive drugs (yes and no), diabetes (yes and no), respiratory disease (yes and no), kidney disease (yes and no), and digestive disease (yes and no).

Supplementary Table 11 **Results from the Cox regression analysis of five favorable individual lifestyle factors and their association with stroke and ischemic stroke**

		Basic model		Full model	
		HR (95% CI)	*P*-value	HR (95% CI)	*P*-value
Stroke	Favorable BMI-WC[a]	0.82 (0.76–0.89)	< 0.000 1	0.92 (0.85–1.00)	0.063 7
	Favorable diet[b]	0.83 (0.75–0.93)	0.000 7	0.86 (0.77–0.96)	0.006 7
	Favorable physical activity[c]	0.91 (0.84–0.98)	0.009 9	0.93 (0.87–1.01)	0.084 1
	Favorable alcohol[d]	0.92 (0.81–1.05)	0.213 7	0.89 (0.79–1.01)	0.083 1
	Favorable smoke[e]	0.82 (0.72–0.94)	0.003 5	0.81 (0.71–0.93)	0.002 1
Ischemic stroke	Favorable BMI-WC[a]	0.77 (0.70–0.84)	< 0.000 1	0.87 (0.80–0.96)	0.004 2
	Favorable diet[b]	0.83 (0.74–0.94)	0.002 1	0.85 (0.75–0.96)	0.007 6
	Favorable physical activity[c]	0.92 (0.84–1.00)	0.046 8	0.95 (0.87–1.04)	0.253 3
	Favorable alcohol[d]	0.94 (0.81–1.08)	0.393 2	0.91 (0.78–1.05)	0.178 8
	Favorable smoke[e]	0.82 (0.71–0.96)	0.011 0	0.81 (0.70–0.94)	0.006 8

Basic model was adjusted for age (linear terms), sex (women and men). The full model was further adjusted for highest education level (no formal school, primary or middle school, and high school and above), household income(< 20 000 RMB/year, 20 000–34 999 RMB/year, and ⩾ 35 000 RMB/year), family history of stroke or heart attack (yes, no, and unknown), duration of sedentary behavior (⩽ 21 h/week, > 21 h/week), usage of antihypertensive drugs (yes and no), diabetes (yes and no), respiratory disease (yes and no), kidney disease (yes and no), and digestive disease (yes and no).

[a]BMI between 18.5 and 27.9 kg/m^2 and a WC < 90 cm (men)/85 cm (women).

[b]Diet score ⩾ 4.

[c]Engaging in an age- (< 50 years, 50–59 years, and ⩾60 years) and sex-specific median or higher level of physical activity.

[d]Nondaily alcohol consumption or daily moderate alcohol consumption.

[e]Nonsmoking or having stopped for reasons other than illness.

Abbreviations: BMI, body mass index; WC, waist circumference.

Supplementary Table 12 **Results from the Cox regression analysis of combined favorable lifestyle factors and their association with stroke and ischemic stroke**

	Quintile 1	Quintile 2	Quintile 3	Quintile 4	*P* trend
Stroke					
Basic model	1.00 (Referent)	0.88 (0.76–1.02)	0.76 (0.66–0.88)	0.64 (0.54–0.75)	< 0.000 1
Full model	1.00 (Referent)	0.90 (0.77–1.04)	0.81 (0.70–0.94)	0.72 (0.61–0.85)	< 0.000 1
Ischemic stroke					
Basic model	1.00 (Referent)	0.84 (0.71–0.99)	0.73 (0.62–0.87)	0.61 (0.51–0.73)	< 0.000 1
Full model	1.00 (Referent)	0.86 (0.73–1.02)	0.79 (0.67–0.93)	0.69 (0.58–0.83)	< 0.000 1

The combined favorable lifestyle score ranges from 0–5. We categorized it into four groups: quintile 1 (combined favorable lifestyle ≤ 1), quintile 2 (combined favorable lifestyle = 2), quintile 3 (combined favorable lifestyle = 3), and quintile 4 (combined favorable lifestyle ≥ 4).

Basic model was adjusted for age (linear terms), sex (women and men). The full model was further adjusted for highest education level (no formal school, primary or middle school, and high school and above), household income(< 20 000 RMB/year, 20 000–34 999 RMB/year, and ≥ 35 000 RMB/year), family history of stroke or heart attack (yes, no, and unknown), duration of sedentary behavior (≤ 21 h/week, > 21 h/week), usage of antihypertensive drugs (yes and no), diabetes (yes and no), respiratory disease (yes and no), kidney disease (yes and no), and digestive disease (yes and no).
